# Association between the reported intensity of an acute symptom at first prehospital assessment and the subsequent outcome: a study on patients with acute chest pain and presumed acute coronary syndrome

**DOI:** 10.1186/s12872-018-0957-3

**Published:** 2018-11-28

**Authors:** Mats Holmberg, Henrik Andersson, Karin Winge, Camilla Lundberg, Thomas Karlsson, Johan Herlitz, Birgitta Wireklint Sundström

**Affiliations:** 1 Department of Ambulance Service, Sörmland County Council, Eskilstuna, Sweden; 2 School of Health, Care and Social Welfare, Mälardalen University, Eskilstuna, Sweden; 3 PreHospen – Centre for Prehospital Research, University of Borås, Borås, Sweden; 4 Faculty of Caring Science, Work Life and Social Welfare, University of Borås, Borås, Sweden; 5 Ambulance Department, South Älvsborgs Hospital, Borås, Sweden; 6 Health Metrics at Sahlgrenska Academy, University of Gothenburg, Gothenburg, Sweden

**Keywords:** Acute coronary syndrome, Ambulance care, Ambulance nurse, Assessment, Chest pain, Patients´ experiences

## Abstract

**Background:**

To decrease the morbidity burden of cardiovascular disease and to avoid the development of potentially preventable complications, early assessment and treatment of acute coronary syndrome (ACS) are important. The aim of this study has therefore been to explore the possible association between patients’ estimated intensity of chest pain when first seen by the ambulance crew in suspected ACS, and the subsequent outcome before and after arrival in hospital.

**Methods:**

Data was collected both prospectively and retrospectively. The inclusion criteria were chest pain raising suspicion of ACS and a reported intensity of pain ≥4 on the visual analogue scale.

**Results:**

All in all, 1603 patients were included in the study. Increased intensity of chest pain was related to: 1) more heart-related complications before hospital admission; 2) a higher proportion of heart failure, anxiety and chest pain after hospital admission; 3) a higher proportion of acute myocardial infarction and 4) a prolonged hospitalisation. However, there was no significant association with mortality neither in 30 days nor in three years. Adjustment for possible confounders including age, a history of smoking and heart failure showed similar results.

**Conclusion:**

The estimated intensity of chest pain reported by the patients on admission by the ambulance team was associated with the risk of complications prior to hospital admission, heart failure, anxiety and chest pain after hospital admission, the final diagnosis and the number of days in hospital.

**Trial registration:**

ClinicalTrials.gov 151:2008/4564 Identifier: NCT00792181. Registred 17 November 2008 ‘retrospectively registered’.

## Background

Early assessment and treatment of acute coronary syndrome (ACS) are major contributors to decreasing the morbidity burden of cardiovascular disease and to avoiding the development of potentially preventable complications [1]. In Sweden every ambulance has to be manned with at least one ambulance nurse (AN) who is a registered nurse with or without specialist training in prehospital emergency care [2]. Ambulance nurses are thus required to assess patients’ symptoms correctly [3, 4], making advanced clinical decisions in order to convey the patients to emergency departments (EDs) or other levels of care [5, 6].

Contradictory suggestions are reported about symptoms that could predict the severity of ACS. European guidelines declare that severe or ongoing chest pain lasting 20 min or more is a symptom indicating high risk of ACS [7]. However it has been established that the severity of chest pain is a poor predictor of imminent complications such as cardiac arrest [8]. In one study, more than 50% of patients with chest pain at the ED were found to have cardiovascular conditions such as myocardial infarction, angina pectoris, pulmonary embolism and heart failure [9]. Others, however, reported that only 9 % of patients admitted to the ED with chest pain had an underlying myocardial infarction [10]. Chest pain symptoms resulting in calling for an ambulance are variable and perceived very differently by different individuals [11].

Earlier studies on patients with acute myocardial infarction have reported a variety of emotional reactions [12] and large variations in patients’ conceptions of the event [13]. It is the patients who provide reliable information about their subjective experiences of symptoms and signs [4]. The localisation of chest pain as reported by patients can vary as well as its intensity, which provides evidence of ANs’ way of taking patients’ subjective experiences into account [14].

These conditions underline the importance of additional and deepened knowledge about early assessment and clinical decisions concerning patients suffering suspected ACS. Reducing time from onset of ACS symptoms to arrival in the coronary care unit at the hospital continues to be a challenge to the healthcare system [15]. For example in the prehospital setting, a more rapid revascularisation has been reported in patients with ST-elevation acute myocardial infarction after prehospital recognition and treatment followed by fast-tracking to a coronary care unit [16].

Experiences from Sweden suggest that more than half of all patients with a final diagnosis of ACS dial 112 for transport by ambulance to hospital. Among these patients a very large proportion has an electrocardiogram (ECG) recorded on scene that often is sent directly to the nearest cardiology department for further evaluation. Among patients with ECG signs of myocardial ischaemia a direct transport to the catheterisation laboratory or coronary care unit is today a routine procedure [16].

The pathophysiology behind the chest pain and the variability in patients’ experiences of chest pain when suffering from ischaemic heart diseases remains obscure. However, in a retrospective study on chest pain in patients admitted to hospital, ambulance patients received the final diagnosis of acute myocardial infarction more often than patients not transported by ambulance [10]. In a retrospective study measuring the intensity of chest pain in the prehospital setting, a shorter prehospital delay was more likely for patients who reported a higher pain intensity than others [17]. Since a shorter prehospital delay has been associated with a higher likelihood of an underlying myocardial infarction [18], these findings suggest that at least some patients intuitively recognise their pain as being of a more severe nature.

However, not much is known on the subject of symptoms such as chest pain, discomfort, anxiety and various other clinical findings from the prehospital setting regarding the predictable severity of ischaemic heart diseases including the risk of complications. Here, there is a gap in knowledge and therefore the aim of this study has been to explore the possible connection between one aspect of chest pain, i.e. the patients’ estimated intensity of chest pain when first seen by the ambulance crew in suspected ACS, and clinical findings and complications before and after arrival in hospital.

## Methods

### Study design and setting

The data for the present study was collected from a Randomised Clinical Trial (RCT no. NCT00792181), here called the main study, which has previously been reported [19–21]. The randomisation was conducted using an envelope method. In the main study patients were allocated to four different treatment alternatives according to a four-factor design. They were: 1) The combined treatment with morphine and midazolam (anxiolytic) by an AN with specialist education in prehospital cardiac care; 2) Treatment with morphine only by an AN with specialist education in prehospital cardiac care; 3) The combined treatment with morphine and midazolam by an AN with no specialist education, and 4) Treatment with morphine only by an AN with no specialist education. The primary aim was to evaluate the effect of the four interventions on the intensity of chest pain before arrival in hospital among patients with chest pain and where the AN had suspected an ACS.

The present study had a prospective randomised and a retrospective observational design. The intensity of chest pain as well as the occurrence of various complications in the prehospital setting were all prospectively reported, whereas data from hospital records was retrospectively reported. All the data was collected in Western Sweden (1.5 million inhabitants) with five ambulance care services, involving 500 ANs, 60 ambulances and one ambulance boat. All the ambulance services participating in the study were staffed with at least one AN. Data collection started in May 2008 and ended in December 2010.

### Measures of pain intensity by the visual analogue scale (VAS)

The VAS has been found to be valid, reliable and appropriate for use in clinical practice, using black lines and a numeric scale [22]. Based on the VAS a Coloured Analogue Scale (CAS) [23] ruler has been developed using gradations in colour and area (and a numerical scale on the reverse side), as well as length, so that the patient can see concretely how different scale positions would reflect different values in their pain intensity. The CAS ruler has equivalent psychometric properties to VAS. However, the CAS ruler was rated as easier to administer and score than the VAS, so it may be more practical for routine clinical use [23]. In addition, the CAS has previously been shown to have psychometric properties for pain measurements in adults [24].

### Study population

All patients were transported by ambulance care services in Western Sweden and participated in the RCT (see above). The intensity of pain (VAS ≥4) was an inclusion criterion for receiving pain treatment in the main study [19]. The inclusion criteria were therefore: complaint of chest pain or discomfort that roused suspicion of ACS and an intensity of pain ≥4 on a VAS, grading from 0 (no pain) to 10 (highest imaginable pain). There was no further definition of chest pain with regard to type and localisation. The assessment of pain took place before the start of pain relieving treatment.

The exclusion criteria were: 1/ Systolic blood pressure < 100 mmHg, 2/ age < 18 years, 3/ under the influence of alcohol, 4/ under the influence of drugs, 5/ benzodiazepine abuse, 6/ dementia, disorientation, 7/ communication problems, 8/ symptoms assessed as being caused by trauma and 9/ secondary transports (in cases where treatment had already been already started).

### Sampling procedure and data collection

Patients were instructed by the AN to use the CAS ruler as follows: “I want you to show your chest pain or discomfort on this scale. The bottom of the scale corresponds to no pain or discomfort. The top corresponds to the most severe pain or discomfort you can imagine. I will move the red line along the scale from the bottom until you say stop”. The CAS ruler was then placed in a vertical position. There were no figures on the side of the instrument that were shown to the patient. By turning the CAS ruler the AN could observe a numeral VAS indicating the level of the patient’s estimated chest pain or discomfort.

### Clinical endpoints

The following complications before and after arrival in hospital were considered as clinical endpoints: heart failure, hypotension, AV-block, bradyarrythmias, supraventricular arrhythmias and ventricular arrhythmias, all requiring treatment. Before arrival in hospital was defined as time from arrival of the ambulance on scene until the time when the ambulance arrived at the hospital whereas after arrival in hospital was defined as time on admission to hospital until time of leaving hospital.

Further clinical endpoints were anxiety and chest pain requiring treatment after hospital admission; mortality before hospital discharge and in 30-days; and final diagnosis and the duration of hospitalisation.

The complications that took place before arrival in hospital were reported prospectively by the AN on duty, whereas complications and other findings that took place after arrival in hospital were reported retrospectively by a few research nurses who collected the information from the hospital records. These records included notes made both by the responsible physician and by the nurse. Information on anxiety was also collected from these notes.

The final diagnosis was assessed according to the ICD code given by the responsible physician and 30-days mortality was assessed according to the Swedish population registry. All deaths in Sweden reach this registry within two weeks after the event.

### Data analyses

For descriptive purposes patients were divided into three groups according to their subjective assessment of chest pain on the arrival of the AN according to the initial value of VAS: 1). 4.00–5.99; 2). 6.00–7.99; 3). ≥8.00. *P*-value calculations were based on the actual values.

### Statistical analysis

For the univariate analyses of association between initial severity of chest pain and clinical findings as well as baseline characteristics, the actual VAS value was used in *p*-value calculations, using the Mann-Whitney U test for dichotomous variables and Spearman’s rank correlation statistic for continuous variables. For calculation of odds ratios with corresponding 95% confidence intervals and *p*-values, logistic regression was used, both unadjusted and when adjusting for potential confounders, defined as baseline characteristics with *p* < 0.20 for association with VAS value. Correspondingly, Spearman’s partial rank correlation was used for adjusted correlation coefficient regarding length of hospitalisation. Thirty days and 3 years mortality was estimated by the Kaplan-Meier method and Cox proportional hazards model was used for calculation of hazard ratios with corresponding confidence intervals and p-values. All tests are two-sided and p-values below 0.05 were considered statistically significant. SAS for Windows version 9.4 was used for the analysis.

## Results

All in all, 1836 patients were included in the main study [19]. Of these, 1767 (96%) patients were eligible for the present study. In 1640 individuals, information on VAS when the ambulance team arrived was received, and of these 1603 had a value of ≥4 at randomisation and constituted the population analysed.

### Age, gender and previous medical history in relation to estimated chest pain

Patients with the most severe chest pain tended to be younger and more frequently to be smokers. Otherwise the estimated intensity of chest pain on the arrival of the ambulance clinicians was not significantly associated with characteristics in terms of distribution of gender and previous history of various diseases (Table 1).

**Table 1:** Age, gender and previous medical history in relation to estimated chest pain

	Intensity of chest pain on arrival of the AN			
	4.00–5.99	6.00–7.99	≥8.00	
	(*n* = 651)	(n = 603)	(*n* = 349)	*p* ^b^
Years of age	72 (62,80)	71 (61,81)	68 (58,78)	0.01
Women (1)^a^	297 (46)	272 (45)	153 (44)	0.70
Previous medical history				
Myocardial infarction (48)	224 (35)	322 (38)	114 (34)	0.63
Angina pectoris (60)	185 (29)	166 (28)	91 (27)	0.57
Heart failure (65)	95 (15)	92 (16)	37 (11)	0.12
Diabetes (40)	121 (19)	128 (22)	60 (18)	0.91
Hypertension (49)	268 (42)	256 (44)	132 (40)	0.65
Chronic obstructive pulmonary disease (42)	50 (8)	58 (10)	31 (9)	0.43
Stroke (41)	66 (10)	55 (9)	35 (10)	0.93
Peripheral artery disease (48)	23 (4)	13 (2)	12 (4)	0.91
Renal disease (43)	47 (7)	29 (5)	26 (8)	0.93
Cancer (41)	67 (11)	61 (10)	34 (10)	0.87
Smoking (356)	85 (17)	84 (18)	66 (24)	0.03

Data presented as median (25th,75th percentile) or number (percentage)

^a^Number of patients with missing information

^b^Actual VAS value used in *p*-value calculation

### Complications prior to hospital admission in relation to estimated chest pain

There was an association between the severity of chest pain and the proportion of hypotension as well as AV-block/bradyarrhythmia requiring treatment. Thus, the risk of these complications increased with increasing severity of pain. Among the patients with the most severe pain the absolute risk was still low, only 2.0% (95% CI 0.8–4.2%) for hypotension and 1.7% (95% CI 0.6–3.8%) for AV- block / bradyarrhythmia. The absolute difference in risk between moderate pain (4–6) and severe pain (> 8) regarding these two complications was 1.4 and 1.6%, respectively. There was no significant association between the estimated intensity of chest pain and other complications before arrival in hospital (Table 2).

**Table 2:** Complications prior to hospital admission in relation to estimated chest pain

	Intensity of pain on arrival of the AN			
	4.00–5.99 (n = 651)	6.00–7.99 (n = 603)	≥8.00 (n = 349)	*p* ^b^
Heart failure requiring treatment (15)^a^	6 (0.9)	5 (0.8)	7 (2.0)	0.11
Hypotension requiring treatment (16)	4 (0.6)	10 (1.7)	7 (2.0)	0.04
AV-block/bradyarrythmias requiring treatment (15)	1 (0.2)	1 (0.2)	6 (1.7)	0.0004
Supraventricular tachyarrythmias requiring treatment (17)	7 (1.1)	5 (0.8)	3 (0.9)	0.21
Ventricular tachyarrythmias requiring treatment (18)	1 (0.2)	1 (0.2)	2 (0.6)	0.41

Data presented as number (percentage)

^a^Number of patients with missing information

^b^Actual VAS value used in *p*-value calculations

### Complications after arrival in hospital in relation to estimated chest pain

Heart failure requiring treatment after arrival in hospital was associated with an increased estimated severity of chest pain; otherwise no significant association was found (Table 3). Among the patients with the most severe pain, the absolute risk of heart failure requiring treatment was 14% (95% CI 11–19%), with an absolute difference in risk between moderate pain and severe pain of 5%.

**Table 3:** Complications after arrival in hospital in relation to estimated chest pain

	Intensity of pain on the arrival of the AN			
	4.00–5.99 (n = 651)	6.00–7.99 (*n* = 603)	≥ 8.00 (n = 349)	*p* ^b^
Heart failure requiring treatment (48)^a^	58 (9.1)	77 (13.1)	48 (14.4)	0.05
Hypotension requiring treatment (48)	27 (4.3)	21 (3.6)	15 (4.5)	0.95
AV-block/bradyarrythmias requiring treatment (48)	11 (1.7)	17 (2.9)	5 (1.5)	0.66
Supraventricular tachyarrythmias requiring treatment (46)	35 (5.5)	31 (5.3)	20 (6.0)	0.71
Ventricular tachyarrythmias requiring treatment (50)	7 (1.1)	5 (0.9)	7 (2.1)	0.37

Data presented as number (percentage)

^a^Number of patients with missing information

^b^Actual VAS value used in p-value calculations

### Clinical findings after admission to hospital in relation to estimated chest pain

There was an association between the estimated severity of chest pain before arrival in hospital and the proportion of patients who experienced anxiety or chest pain requiring treatment after arrival in hospital. Among the patients with the most severe pain the absolute risk of suffering from anxiety which required treatment, was 15% (95% CI 11–19%) and of symptoms of chest pain requiring treatment 42% (95% CI 37–48%). The absolute difference in risk between moderate pain and severe pain regarding these two findings were 5 and 10%, respectively.

There was also an association between the estimated intensity of chest pain and the proportion of patients who received a final diagnosis of acute myocardial infarction. Among patients with the most severe pain the absolute risk was 33% (95% CI 28–39%), with an absolute difference in risk between moderate pain and severe pain of 11%.

Additionally, more severe chest pain was associated with an increase in the number of days that the patients spent in hospital (Table 4).

**Table 4:** Clinical findings after admission to hospital in relation to estimated chest pain

	Intensity of pain on arrival of the AN			
	4.00–5.99 (n = 651)	6.00–7.99 (n = 603)	≥8.00 (n = 349)	*p* ^b^
Anxiety requiring treatment (%) (56)^a^	58 (9.2)	62 (10.6)	48 (14.5)	0.009
Pain requiring treatment (%) (60)	203 (32.1)	241 (41.2)	138 (42.5)	< 0.0001
Final diagnosis (%) (97)				
Myocardial infarction	137 (22.4)	136 (23.9)	108 (33.1)	0.004
Angina pectoris	65 (10.6)	62 (10.9)	28 (8.6)	0.46
Myocardial infarction or angina pectoris	202 (33.0)	198 (34.9)	136 (41.7)	0.03
Survival at discharge or not admitted to a hospital ward (%) (37)	627 (98.1)	582 (98.5)	333 (99.1)	0.11
Hospitalisation (median (25th,75th percentile) number of days) ^c^	2 (2,5)	3 (2,6)	3 (2,6)	< 0.0001

Data presented as median (25th,75th percentile) or number (percentage)

^a^Number of patients with missing information

^b^Actual VAS value used in p-value calculations

^c^Calculated from the time of calling ambulance service

### Strength of the association

The strength of the association is shown in Table 5, in terms of odds ratios, between the estimated intensity of the pain/discomfort and the risk of various complications for those complications where a significant association were found in univariate analysis, both unadjusted and adjusted for the potential confounders age, history of smoking and history of heart failure.

**Table 5:** Strength of the association

	OR (95% CI)^a^	*p*
Before arrival in hospital		
Hypotension requiring treatment		
unadjusted	1.31 (1.02,1.69)	0.04
adjusted^b^	1.22 (0.92,1.62)	0.16
AV-block/bradyarrhythmias requiring treatment		
unadjusted	2.23 (1.44,3.45)	0.0003
adjusted^b^	2.52 (1.39,4.57)	0.16
After arrival in hospital		
Heart failure requiring treatment		
unadjusted	1.11 (1.01,1.23)	0.03
adjusted^b^	1.26 (1.12,1.43)	0.0002
Anxiety requiring treatment		
unadjusted	1.14 (1.04,1.26)	0.008
adjusted^b^	1.18 (1.05,1.33)	0.005
Pain requiring treatment		
unadjusted	1.14 (1.07,1.22)	< 0.0001
adjusted^b^	1.17 (1.08,1.26)	< 0.0001
Final diagnosis of AMI		
unadjusted	1.14 (1.06,1.23)	0.0003
adjusted^b^	1.13 (1.04,1.23)	0.003
Final diagnosis of AMI or angina pectoris		
unadjusted	1.09 (1.02,1.17)	0.009
adjusted^b^	1.11 (1.03,1.20)	0.007
Length of hospitalisation		
unadjusted	*r* = 0.11	< 0.0001
adjusted^b^	*r* = 0.14	< 0.0001

^a^odds ratio per unit in the VAS scale with corresponding 95% confidence interval, except for length of hospitalization, where Spearman’s rank correlation between VAS value and number of days are given

^b^adjusted for age, a history of smoking and a history of heart failure

### Intensity of pain and risk of death

**Fig. 1:**
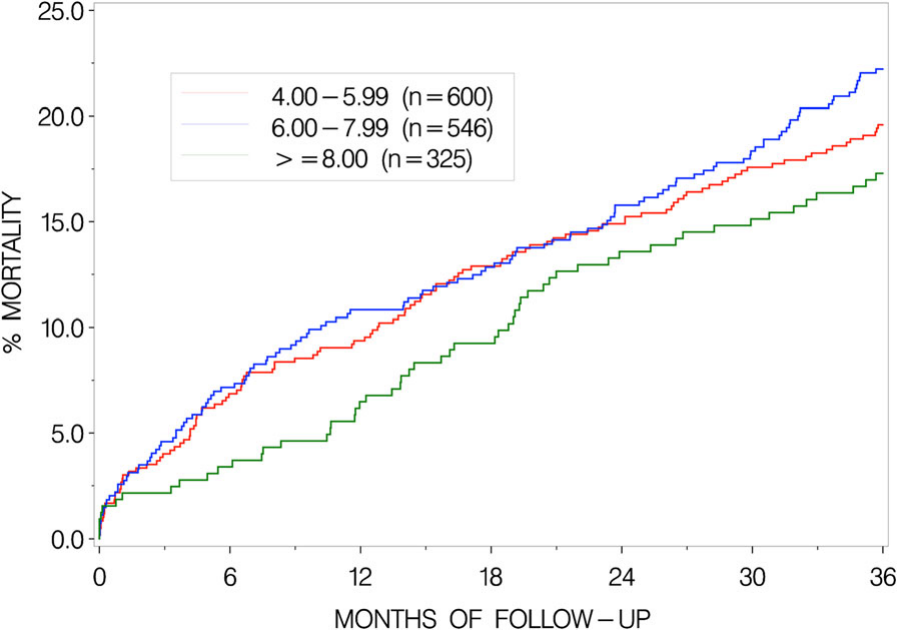
The cumulative mortality during 30 days and 3 years after hospital admission in the three pain level groups

In Fig. 1 is shown the cumulative mortality during 30 days and 3 years, respectively, after hospital admission in the three pain level groups. No association was found neither in univariate analysis nor when adjustment for the three possible confounders was performed, neither with regard to 30-day mortality (unadjusted hazard ratio (HR) per unit of the VAS scale 0.88, 95% confidence interval (CI) 0.70,1.10, *p* = 0.25; adjusted for confounders 0.96 (0.74,1.24), *p* = 0.73) nor to 3-year mortality (unadjusted 0.96 (0.89,1.03), *p* = 0.27; adjusted 0.98 (0.89,1.08), *p* = 0.65).

## Discussion

This is to the best of our knowledge the largest study reporting on the connection between the intensity of chest pain according to subjective assessment on ambulance arrival and clinical findings before and after arrival in hospital among patients with acute chest pain raising suspicion of ACS.

The principal research question being addressed is the degree of association between the patient-reported intensity of an acute symptom at an early assessment and the subsequent outcome. We found significant but mostly weak associations between the patients’ estimated severity of chest pain on admission of the ambulance clinicians and a number of indicators reflecting the severity of the disease. These findings are new and suggest that the severity of chest pain as assessed by patients using the VAS may give some indirect indications of the severity of the disease underlying the symptom presentation in prehospital assessments. Some of the study’s outcomes are strictly time dependent conditions (e.g. myocardial infarction) and some are not (e.g. angina pectoris). Our findings could be related to a Norwegian study showing that a majority of ambulance patients with chest pain were admitted for further assessment in hospital, but only a quarter were assessed in the prehospital setting as having a severe illness [25]. These facts together draw attention to the importance of ANs’ ability to make prehospital assessments using the patients’ experience of pain as important information. However, pain may be understood as a complex phenomenon not fully encompassed by measuring pain intensity using the VAS.

The present results show a connection between estimated intensity of chest pain and AV block/bradyarrhythmias in the prehospital setting. In general, the proportion of various complications prior to hospital admission was very low, contributing to the failure of finding any significant association between the reported intensity of chest pain and the majority of complications prior to arrival to hospital. The ambulance care organisation is affected by an ambition to reduce time delay, which may lead to stress with a detrimental effect on patient care [26]. However, rapid transport to hospital care allows less time for patients’ clinical complications to develop. Today guidelines are considered important to help accomplish fast prehospital assessments of patients with ST-elevation ACS in order to reduce time delay to in-hospital treatment [3]. However, the present results may support the development of prehospital guidelines to assist assessment of ACS without ST-elevation as well [27].

After admission to hospital, the proportion of various haemodynamic complications as well as supraventricular arrhythmias increased, whereas ventricular arrhythmias and bradyarrhythmias remained infrequent. No significant association between the early assessment of the intensity of chest pain in the prehospital setting and these complications was found with exception for heart failure. Hence this may indicate a weak association between the early intensity of chest pain prior to arrival to hospital and the severity of the disease. Galinski et al. found no significant difference regarding the severity of pain between ambulance transported patients with acute myocardial infarction and no myocardial infarction [28]. They argued for chest pain severity as a useless factor for distinguishing acute myocardial infarctions from other conditions. In addition, Thuresson et al. found that only half of the patients with ST-elevation ACS reported that chest pain appeared suddenly and rapidly reached a high intensity [29]. However, this contrasted with our findings of a significant association between estimated intensity of chest pain and the proportion of a final diagnosis of acute myocardial infarction. This is in agreement with a previous study showing that when the dispatchers scored more severe chest pain based on patient interviews, then the likelihood of acute myocardial infarction was much higher [30].Their results indicated that the severity of the pain was one of the most important questions to address in order to optimise the priority at the dispatch centre. However, this finding does not exclude the risk of an underlying myocardial infarction or complications even among patients with a lower intensity of pain.

The present finding of a significant association between estimated intensity of chest pain and the duration of hospitalisation may be explained by a higher proportion of acute myocardial infarction with increasing severity of pain. This is in line with an earlier study by Goodacre et al., showing pain as a predictor of acute myocardial infarction and ACS in patients with normal or non-diagnostic ECG [31]. Patients who did not have relief of chest pain after 15 min and on admission to hospital constituted a minority in the present study.

Our results showed an association between the reported initial intensity of chest pain and anxiety as well as chest pain after admission to hospital. This may correspond to myocardial infarction patients’ vulnerability due to lacking control of the situation, experiencing anxiety [13] and fearing death as a possible outcome [32]. One factor causing anxiety after arrival in hospital may be the reduced presence of medical staff in the ED compared with the ambulance environment [33], resulting in a feeling of isolation and insecurity [34]. The present findings corroborate this interpretation and may indicate the importance of taking the patients’ whole life situation into account when assessing their physical disorder [4]. Forslund et al. found in a study on ambulance patients that acute chest pain is a subjective experience that accompanies fear and anxiety [35].

### Strexngths and limitations

This is a study with a very large sample size, which must be seen as a strength. The pain was prospectively reported based on the patients’ subjective assessment. The measurement was made early in the course before the administration of pain treatment. Furthermore, in the statistical analyses adjustment were made for possible confounders in terms of age and previous history.

However, this method for estimation of the intensity of chest pain has several limitations. The data received via VAS is at an ordinal level dependent on the patients’ subjective pain assessment. However, the fact that VAS does not collect information about the pain quality, duration or localisation, must be regarded as a limitation. This study only included patients with an estimated initial VAS of ≥4. Therefore the full spectrum of patients’ chest pain experience was not included in the analyses, which may have led to missed cases and potential selection bias error. This fact might underscore the true association between the intensity of chest pain and the subsequent outcome. One cannot exclude the possibility that other types of symptoms, e.g. dyspnea, nausea and vomiting might be equally important in the evaluation of the association between the initial intensity of chest pain and the subsequent outcome. This emphasises the importance of studying and developing methods for chest pain assessments that include other symptoms as well, such as pain quality, duration and localisation.

Furthermore, patients with a systolic blood pressure of < 100 mmHg were also excluded from participation. Such patients often have indications of a compromised circulation with an unstable clinical condition. Thus, the study cohort focuses on patients with a more stable clinical condition. We report on the intensity of symptoms at the time of admission by the ambulance clinicians and not at the time of symptom onset when symptoms are most probably even more severe.

All the patients included in these analyses participated in a randomised study which has been reported earlier [19–21]. Hence, the patients who participated in the study had to give informed consent prior to hospital admission. However, this may have resulted in a selection bias and patients with the most severe symptoms were most probably not always included in the present study. Furthermore, there is no information on the response rate, i.e. the proportion of patients who did not wish to participate in the study. The possible selection bias constitutes a limitation in this study and signifies a major challenge when conducting studies on severely ill patients when informed consent is required.

There was no external monitor who validated the quality of the data with regard to clinical endpoints such as heart failure and arrhythmias. However, since only conditions that required treatment were considered we assume a high validity. In Swedish healthcare, all medications that are given by healthcare providers must be carefully recorded.

The lack of association between the intensity pain and the risk of death particularly during long term must be interpreted with caution. A number of unmeasured confounders are likely to affect this association, of which revascularization may be the most important one. Furthermore may the inclusion of patients with less time dependent conditions such as chronic angina pectoris be a confounder. In such conditions may the association between the intensity of pain an outcome be different as compared with myocardial infarction.

Finally, the last patient was included in the study seven years ago. However, during these seven years there has been no major change in the prehospital assessment of care among patients with chest pain either with regard to treatment or care in the region in which this study took place.

## Conclusions

In patients with chest pain raising suspicion of ACS on admission by the ambulance clinicians and then transported by ambulance, the estimated intensity of chest pain was associated with complications prior to hospital admission, heart failure, anxiety and chest pain after arrival in hospital, final diagnosis and the number of days in hospital. One implication of this study might be that when ANs suspect ACS the likelihood of an underlying acute myocardial infarction is somewhat higher if the patient in addition scores high on VAS.

However, the association between early assessment and the subsequent outcome was relatively weak as patients with lower intensity (VAS < 4) of chest pain were not included in the study and therefore cannot be commented on. Hence, this study provides knowledge about patients reporting prehospital chest pain measuring VAS 4 or higher. In order to generate a wider picture of the association between reported intensity of prehospital chest pain and the subsequent outcome, studies on patients reporting lower intensity of pain are important as a complement to the present study. In addition, risk scores for patients with acute chest pain might be developed specifically for the prehospital setting in which the intensity of the initial chest pain could be one of a number of different aspects of the clinical picture.

### Ethical approval and consent to participate

Ethical considerations were taken into account in line with the Declaration of Helsinki [36]. The patients who were required to give written informed consent were provided with both written and oral information about the study, emphasising confidentiality. Withdrawal would not affect their current ambulance care or their subsequent care in hospital and was possible at any time without stating any reason. Ethical approval was obtained from the Research Ethics Committee in Gothenburg (registration number 022–08).

## Abbreviations

ACS: Acute Coronary Syndrome
AN: Ambulance Nurse
CAS: Coloured Analogue Scale
ECG: Electrocardiogram
ED: Emergency Departments
VAS: Visual Analogue Scale
